# An Appraisal of the Clinical Features of Pediatric Enteric Fever: Systematic Review and Meta-analysis of the Age-Stratified Disease Occurrence

**DOI:** 10.1093/cid/cix229

**Published:** 2017-03-27

**Authors:** Carl Britto, Andrew J. Pollard, Merryn Voysey, Christoph J. Blohmke

**Affiliations:** 1 Oxford Vaccine Group, Department of Paediatrics, University of Oxford and the National Institute for Health Research Oxford Biomedical Research Centre and; 2 Nuffield Department of Primary Care Health Sciences, University of Oxford, United Kingdom

**Keywords:** pediatric, typhoid, paratyphoid, enteric fever, clinical.

## Abstract

Children bear a substantial proportion of the enteric fever disease burden in endemic areas. Controversy persists regarding which age groups are most affected, leading to uncertainty about optimal intervention strategies. We performed a systematic review and meta-analysis of studies in Asia and Africa to compare the relative proportion of children with enteric fever in the age groups <5 years, 5–9 years, and 10–14 years. Overall, studies conducted in Africa showed a relatively smaller occurrence of disease in the youngest age group, whereas in Asia the picture was more mixed with a very large degree of heterogeneity in estimates. The clinical features of enteric fever reviewed here differ between younger and older children and adults, likely leading to further uncertainty over disease burden. It is evident from our review that preschool children and infants also contribute a significant proportion of disease burden but have not been adequately targeted via vaccination programs, which have been focusing primarily on school-based vaccination campaigns.

## Summary

Our systematic review and meta-analysis compares the relative age-stratified proportion of children with enteric fever in Asia and Africa and provides a review of clinical features of pediatric enteric fever in comparison with adult disease.

Enteric fever, caused by *Salmonella enterica* subspecies *enterica* serovars Typhi, Paratyphi A, Paratyphi B, and Paratyphi C, is a major cause of morbidity for human populations in affected regions of the world. Currently, it is estimated that there are >26 million patients with a blood culture positive for enteric fever annually with a 1% fatality rate [1]. The majority of existing epidemiological evidence comes from studies in adult populations. There are conflicting opinions about the rate of disease in young children, especially infants [2–7], even though a substantial burden of disease is suffered by young children in endemic regions [2, 3].

The emergence of resistant haplotypes of *Salmonella* makes control via vaccination an urgent priority. For more than a decade, immunization efforts in endemic areas have been focused on school vaccination campaigns [4]. Two licensed typhoid fever vaccines, the oral Ty21a vaccine and the parenteral Vi polysaccharide (ViPS) vaccine, are currently available, both of which have limited use in preschool children due to the mode of administration as capsules via the per oral route (Ty21a), or an inferior immune response in children <2 years of age (ViPS) [5]. Promising conjugate vaccines are on the horizon, but the immunization strategy and the target population for these vaccines are yet to be outlined, as current epidemiological evidence is not adequately informative regarding sources of infection [6], role of chronic carriers [7], and the programmatic effectiveness of conjugate vaccines [4].

## SYSTEMATIC REVIEW AND META-ANALYSIS COMPARING DISEASE OCCURRENCE BETWEEN AGE GROUPS

### Data Input and Analysis

**Figure 1.**
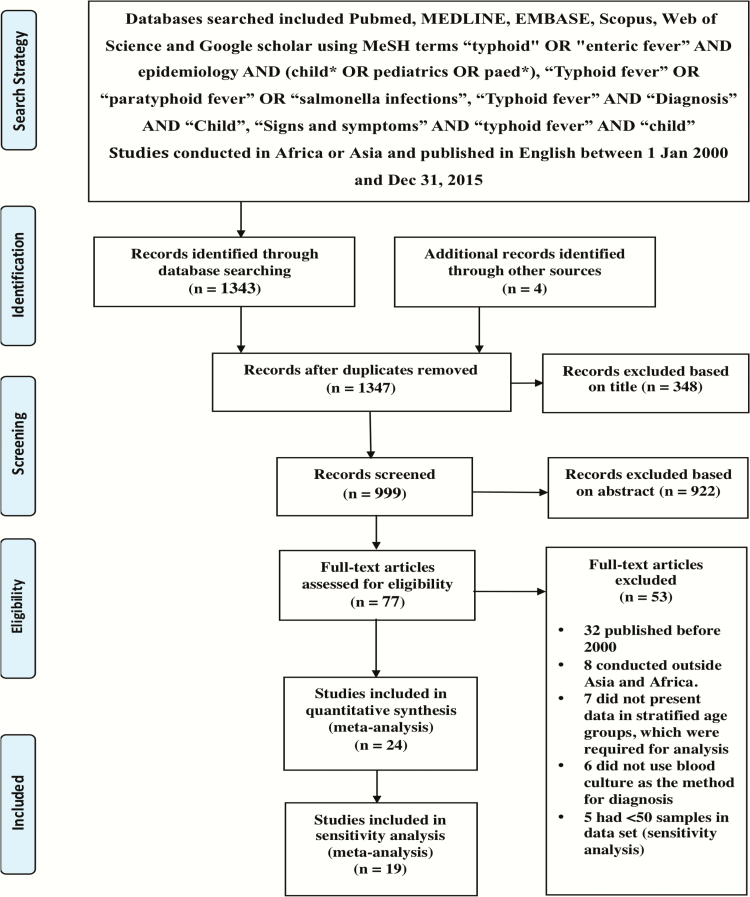
Search strategy and characteristics of included studies in the meta-analysis.

We conducted a systematic review and meta-analysis to characterize the age distribution of pediatric enteric fever in Africa and Asia. A case of enteric fever, for the purpose of this review, was defined as a child with a blood culture yielding *S*. Typhi or *S*. Paratyphi. Full details of the search strategy and methodology are presented in (Figure 1) and the Supplementary Data.

### Results and Discussion

**Figure 2.**
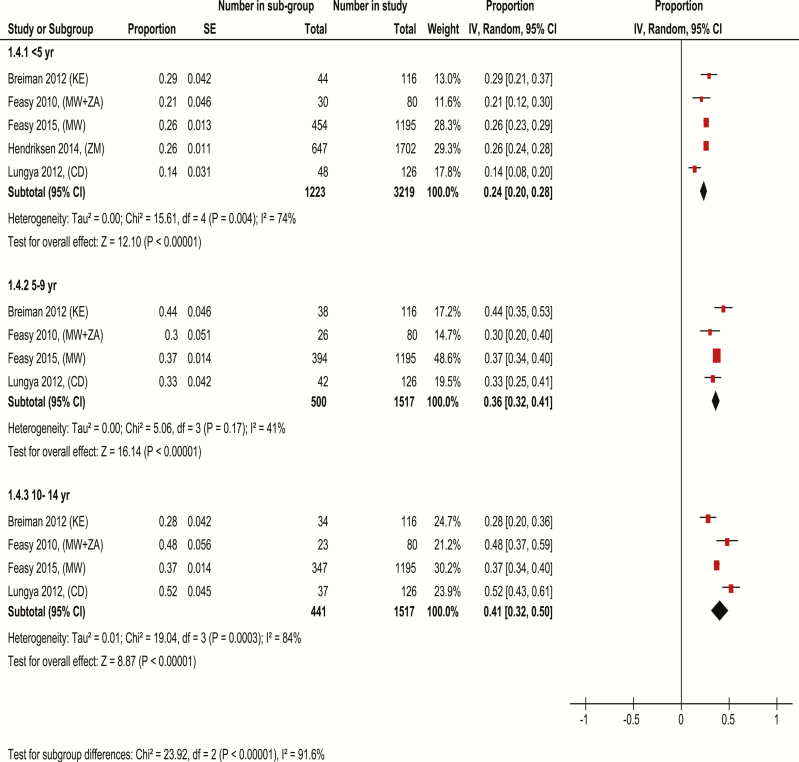
Meta-analysis comparing the age-stratified prevalence of pediatric enteric fever cases in Africa. Studies in the meta-analysis are identified based on author name, year of publication with country codes in parenthesis (country codes used are those supplied by the International Organization for Standardization). Studies with ≤50 cases were excluded. Proportion is (number of children in age group / number of children in the study aged 0–14 years). Proportions are standardized according to population age distributions for the 3 age categories. The analysis was done using random-effects model. Abbreviations: CD, Democratic Republic of Congo; CI, confidence interval; IV, inverse variance; KE, Kenya; MW, Malawi; SE, standard error; ZA, Zimbabwe; ZM, Zambia.

In studies conducted in Africa, which had at least 50 cases, the prevalence of enteric fever in children increased with age. Overall, the smallest proportion of cases (24%) was observed in those aged <5 years, followed by 36% in those 5–9 years of age, and 41% in the older age group. Substantial heterogeneity existed between studies in the youngest and oldest age groups. Estimates in those aged <5 years ranged from 14% to 29%, compared with 30%–44% in those 5–9 years of age and 28%–52% in those 10–14 years of age. Thus, even in the presence of statistical heterogeneity, it would appear that younger children contribute a smaller proportion of pediatric enteric fever in Africa (Figure 2).

The pattern of disease and epidemiological features of pediatric *Salmonella* infections in Africa are dominated by invasive nontyphoidal *Salmonella* and *S.* Typhi infections with negligible prevalence of *S*. Paratyphi. The emergence of multidrug-resistant (MDR) strains of *S.* Typhi, in particular the H58 clone, has resulted in a sharp increase in disease occurrence in East and Southeast Africa [8-11]. For instance, disease prevalence has increased 5-fold over the last 3 years in Malawi primarily due to the arrival of MDR strains [12], with children contributing to >70% of cases in the community [9]. *Salmonella* Typhi strains with reduced susceptibility toward ciprofloxacin are highly prevalent in Kenya but not yet uniformly present across the rest of sub-Saharan Africa [13]. Most of these surveillance data capture inpatient burden, which reflects severe disease. A large proportion of outpatient cases may thus go unobserved, and this is particularly relevant in enteric fever where pediatric outpatients are 66% more likely to have *S*. Typhi in the bloodstream than adult outpatients in sub-Saharan Africa [14].

**Figure 3.**
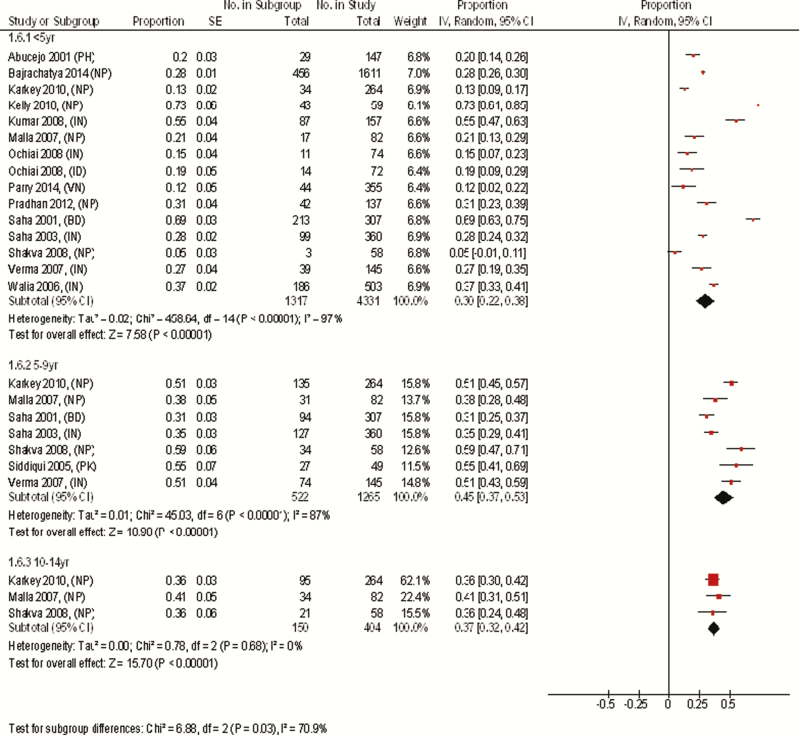
Meta-analysis comparing the age-stratified prevalence of pediatric enteric fever cases in Asia. Studies in the meta-analysis are identified based on author name, year of publication with country codes in parenthesis (country codes used are those supplied by the International Standardization Organisation). Studies with ≤50 cases were excluded. Proportion is (number of children in age group / number of children in the study aged 0–14 years). Proportions are standardized according to population age distributions for the 3 age categories. The analysis was done using random-effects model. Abbreviations: BD, Bangladesh; CI, confidence interval; ID, Indonesia; IN, India; IV, inverse variance; NP, Nepal; PH, Philippines; PK, Pakistan; SE, standard error; VN, Vietnam.

In studies conducted in Asia that included at least 50 participants, the overall disease occurrence in those aged <5 years (30%) was lowest among the 3 age groups but, unlike in Africa, the highest prevalence was seen in those aged 5–9 years (45%) followed by those aged 10–14 years (37%). However, estimates for all age groups showed substantial heterogeneity, which was most notable in those <5 years of age, making comparisons between summary estimates in each age group problematic. The proportion of cases in each study in those aged <5 years ranged from 5% to 73%. Of the 14 studies included in the analysis of the under-5 age group, 3 studies [15–17] estimated that <15% of disease was in this age group, whereas 3 studies [18–20] estimated that more than half the disease occurrence was in this age group (Figure 3). These vastly contradictory findings illustrate the difficulty of diagnosis of enteric fever in children and thus the lack of expert consensus on this topic. Sources of variability in diagnosis among younger children could be related to the high numbers of other nonspecific illnesses in this age group, difficulty in obtaining adequate volumes of blood for culture, lower rates of exposure, and protective effect of breastfeeding. Other potential sources of heterogeneity include study design, the duration of follow-up, diagnostics used, and the possible use of vaccines. We investigated graphically the possible study-specific sources of heterogeneity in those aged <5 years, including prospective or retrospective data collection, community or hospital settings, and duration of follow-up and study size. No strong associations were observed; however, there appeared to be a greater degree of variability in smaller studies than larger ones (Supplementary Figures 3A–C).

A large part of the current understanding of enteric fever has come through studies and surveillance sites in South and Southeast Asia [21–25], where accumulating evidence indicates a substantial burden of pediatric enteric fever. Settings with an incidence of >100/100000 cases per year are considered high-incidence settings, whereas those between 10–100/100000 and <10/100000 cases per year are medium- and low-incidence settings, respectively [26]. Most active surveillance sites in South Asia are high-incidence settings [2, 27–29], with some sites as high as 573/100000 per year [29] in younger pediatric populations. Disease rates in the control arm of cluster-randomized trials also provide estimates of disease incidence among children in the population under survey. Data from Karachi, evaluating the effectiveness of the ViPS vaccine, revealed incidence rates in the control arm of 230/100000 and 190/100000 person-years (PY) in those aged 2–4 years and 5–16 years, respectively, while data from a ViPS vaccine trial in Kolkata reported an incidence of 354/100000 PY and 167/100000 PY in those aged 2–4 years and 5–16 years, respectively [30, 31]. Paratyphoid fever in the pediatric populations occurs mainly in older children and accounts for a lower proportion of enteric fever cases in South Asia [32]. Incidence estimates of *S*. Paratyphi A infection in children are similar to those seen in adults, ranging between 51 and 76 cases/100000 PY. *Salmonella* Paratyphi B and C are rare and only seen sporadically [32]. Interestingly, some evidence suggests that a relatively higher incidence of paratyphoid fever may follow ViPS vaccine introduction [32]. Evidence of cross-protection with the available typhoid vaccines to *S*. Paratyphi B has been demonstrated with the Ty21a vaccine, but there is no strong evidence with respect to *S*. Paratyphi A cross-protection [32, 33]. China, particularly the East region, accounts for the highest burden of paratyphoid fever, while data from India demonstrated considerable heterogeneity between regions with hospital-based data indicating higher proportions of paratyphoid from the West and the South compared with the North and the East [32]. Disease prevalence from Pakistan seemed to mirror the pattern seen in North and East India [32]. It is currently difficult to assess whether the occurrence of pediatric enteric fever in Asia is increasing or decreasing, as there are no repeat studies done in areas of prior disease estimation.

School-aged children >5 years of age have consistently been reported to have high rates of enteric fever, with incidence rates up to 4 times higher than adult populations [29]. Nevertheless, it is becoming increasingly evident that preschool children are also substantially affected, with a surveillance site in Bangladesh reporting an 8.9 times higher likelihood of preschool children acquiring enteric fever than older children and adults [34]. It should, however, be borne in mind that this discrepancy in disease burden may be due to the implementation of school-based vaccination programs in some areas, which is reflected in the lower disease burden among school-aged children in some reports [2]. We attempted to further stratify the age group <5 years, to gauge disease proportion in <2 years in relation to the older age groups. However, this was not possible as most studies did not report the disease prevalence in this age group in relation to older age groups. Only 2 studies [35, 36] reported blood culture volume adequacy, and no studies objectively reported contamination or antimicrobial exposure prior to culture, which contribute to surveillance artefact and underascertainment.

### Inference

The burden of enteric fever in children is difficult to measure, particularly with respect to which age group bears the brunt of the burden. Some studies report an equal occurrence among preschool and older children whereas others report a significantly higher burden in the preschool age group. It should be noted that school-based vaccination programs have been implemented erratically in South Asia, which may account for varied disease prevalence in the school-aged children in Asia.

## CLINICAL FEATURES AND COMPLICATIONS

The clinical features of pediatric enteric fever are nonspecific and overlap with a variety of infective etiologies in endemic settings [3, 37]. The proportion of cases clinical suspected with enteric fever may be as low as 4% at initial presentation in ultimately blood culture–confirmed cases in children [38]. Clinical features, complications, and outcomes differ between adults and children; even among children, differences exist between infants and older children as well as between children in Africa and Asia [3]. A recent systematic review of clinical and laboratory features of enteric fever indicated that the risk of mortality from enteric fever is 4 times higher in children <5 years compared with those >5 years of age [3].

### Generalized Systemic Features

A typical febrile response usually occurs between 5 and 15 days after exposure to the organism as demonstrated in the challenge model in adult subjects [39]. Evidence from a recently published systematic review reported that fever is a consistent feature of pediatric enteric fever, in 97%–100% of cases [3], not uncommonly being the sole manifestation, presenting as a fever of unknown origin [40] with inconclusive laboratory results. Conversely, younger children with enteric fever may sometimes present with hypothermia [Supplementary Reference 41]. The widespread and poorly regulated use of antibiotics and antipyretics in low- to middle-income country settings not only confounds the clinical picture but also serves for the origin and propagation of MDR strains of *Salmonella*. If untreated, the febrile response reaches its peak in the second week of illness in a characteristic “stepladder” pattern [Supplementary Reference 42]. Chills and rigors are 4 times more common in adults than in children [3]. Relative bradycardia, a classical finding in enteric fever, is identifiable in 11%–30% of children [Supplementary References 43–47] and appears to be 15 times more likely to occur in African children than the children in Asia [3].

### Gastrointestinal Features

Gastrointestinal (GI) manifestations of acute enteric fever are variable in children. Diarrhea is seen >2.5 times more commonly in enteric fever affected infants than in older children and adults [3; Supplementary References 37, 41, and 48]. A lesser proportion of children may have constipation, which has been associated with disease relapse in children infected with MDR strains, according to observations from a single-center study over a 15-year period [Supplementary Reference 49]. GI bleeding and perforation, though rare, have been known to occur in children and are 9 times higher in hospital-based studies than community-based settings [3], possibly reflecting a sampling bias of severe cases being referred to hospital from the community. Predictors of death due to perforation in children include older age, high temperature, postoperative anastomotic leak, and fecal fistula [Supplementary References 50 and 51]. A robust immune response coupled with well-primed Peyers patches are main requirements for these complications, which increase with the increasing age of the child, and this is possibly why severe complications are seldom seen in the younger age groups [Supplementary References 48, 50, and 52]. Paralytic ileus is more commonly observed in infants and young children [Supplementary References 53–55] being 7 times more likely in African children than their Asian counterparts, according to Azmatullah et al [3]. Abdominal pain and nausea, which are commonly seen in adults, are also seen in older children but are difficult to determine in young children.

### Neurological Features

The occurrence of seizures in enteric fever is more common in children than adults [Supplementary Reference 48]. The high temperatures associated with enteric fever induce febrile seizures in susceptible children between 6 months and 5 years of age [Supplementary Reference 56]. The occurrence of febrile seizures may account for the higher seizure rate described in children. It is not clear whether *Salmonella* has a direct effect on the central nervous system, as the bacteria are seldom isolated from cerebrospinal fluid during lumbar puncture [Supplementary Reference 57]. Nevertheless, the toxin released by the bacteria may elicit cortical irritation. The typhoid toxin only binds to mammalian cell membranes, made up of glycoproteins containing Neu5Ac-terminated glycans [Supplementary Reference 58], which are also found in gangliosides that are components of the neuronal cell membrane in the brain [Supplementary Reference 59]. The toxin could therefore cause membrane depolarization by presumably interfering with the voltage-gated sodium channels. Further work needs to be done to establish the mechanism at the cell membrane level; however, a distinct frontal intermittent rhythmic delta activity pattern on electroencephalogram (EEG) has been reported in *Salmonella* encephalopathy [Supplementary Reference 60]. This EEG pattern has been associated with the manifestation of seizures [Supplementary Reference 61]. Secondary causes such as hyponatremia and hypoglycemia due to salt and water loss from the gut may be contributing factors. Other neurologic complications, rarely encountered [37] but predominantly described in the pediatric age group, include acute cerebellar ataxia, sinus thrombosis, meningism, cerebritis, pseudo tumor cerebri, encephalopathy, brain abscesses, and Guillain-Barré syndrome [Supplementary References 62–79]. Neuropsychiatric changes, delirium, insomnia, and coma are also described in children but are more frequent in adults [Supplementary Reference 48].

### Hepatosplenic Features

A clinically tender hepatomegaly and splenomegaly are seen in up to 85% and 90%, respectively, of pediatric enteric fever cases [Supplementary References 44 and 80]. Typhoid hepatitis or “hepatitis typhosa” is more frequently seen in younger children but assumes importance as it mimics acute viral hepatitis in the tropics and is likely to either be immunologically mediated or due to the direct effects of the typhoid toxin on the hepatocytes [Supplementary Reference 81]. Acalculous cholecystitis has also been described in brief reports and occurs predominantly in younger children [3, Supplementary References 53–55]. Hepatic and splenic abscesses with a subsequent splenic abscess rupture are more commonly encountered in younger children, the immunosuppressed, and those with hemoglobinopathies [Supplementary Reference 55]. Splenic rupture, a devastating complication, though uncommon in children, is particularly associated with MDR infection [Supplementary References 82 and 83], although it is not clear whether this occurs due to treatment failure or virulence of the pathogen.

### Cardiopulmonary Features

Case reports and expert reviews describe cardiac complications such as myocarditis, endocarditis, pericarditis, and pericardial effusion, which are more common in older children and adults with risk factors such as congenital heart disease, rheumatic heart disease, and valvular defects [Supplementary Reference 55]. Respiratory symptoms may dominate the clinical picture of pediatric enteric fever, with cough being the most common manifestation seen in up to 72% of cases [Supplementary Reference 84]. A clinical picture of bronchopneumonia, one of the respiratory complications of enteric fever, also occurs occasionally [Supplementary Reference 53] and is twice as common in children than in adults [3]. A clinical picture of reactive airway disease with auscultatory rhonchi and occasional crepitations may be noted in younger children [Supplementary Reference 47]. Risk factors for complications such as pleural effusion, empyema, and bronchopleural fistulas [Supplementary Reference 55], though seldom reported in children, may occur in those with previous respiratory infections, sickle cell anemia, and immunosuppression [Supplementary Reference 55].

### Hematological Features

The transient pancytopenia seen in acute illness may, in part, be explained by seeding of *Salmonella* to the bone marrow [37]. A systematic review suggests that during an episode of enteric fever, children in Africa have a 5 times greater risk of severe anemia and a 15 times greater risk of thrombocytopenia than children in Asia [3]. Further evidence is required to confirm whether *S*. Typhi causes these hematological abnormalities independently and to what extent the host genetics play a role. As a facultative intracellular gram-negative organism, *Salmonella* induces apoptosis in the macrophages that it infects, contributing to the leukopenia [Supplementary Reference 85]. Eosinopenia in particular is an important indicator of disease severity [Supplementary Reference 86] that may be seen in up to 70% of children with enteric fever and has been well documented in adult patients as well as in the human challenge model [Supplementary Reference 39]. On the other hand, and contrary to the leukopenia seen in adults, a peculiar finding in children is the relatively common occurrence of leukocytosis or the presence of a leukocyte count within the normal range [9, 17]. In fact, leukocytosis is almost 3 times more likely to occur in children than in adults, while children under 5 are >4 times more likely to have an elevated leukocyte count than older children. Butler et al observed a strong association of a leukemoid response with shigellosis [Supplementary Reference 87], which was possibly induced by the lipopolysaccharide [Supplementary Reference 88] present in gram-negative bacilli, which might also be the case in enteric fever. Rare hematologic complications reported in enteric fever include disseminated intravascular coagulation, hemophagocytosis, bone marrow suppression, and bone marrow granulomata, all of which are more commonly described in adults than in children [3; Supplementary References 47 and 55].

## CONCLUSIONS

In this review and meta-analysis, we have identified a substantial burden of enteric fever in childhood in the disease-affected regions of the world. We highlighted differences in clinical appearance of the disease, especially in the youngest children, and the likely hidden burden of missed clinical cases. A point-of-care diagnostic with a strong positive predictive value, which improves pediatric enteric fever diagnosis, will dissuade empiric antibiotic treatment, which is implicated in the development of antibiotic resistance. As antibiotic resistance spreads, there is real hope that deployment of typhoid conjugate vaccines in the years ahead could catalyze the eradication of typhoid in conjunction with improving access to clean water.

## Supplementary Data

Supplementary materials are available at *Clinical Infectious Diseases* online. Consisting of data provided by the authors to benefit the reader, the posted materials are not copyedited and are the sole responsibility of the authors, so questions or comments should be addressed to the corresponding author.

## Supplementary Material

Supplememtary_1

Supplementary_2

Supplementary_3a

Supplementary_3b

Supplementary_3c

Supplementary-material_2

## Notes

***Author contributions.*** C. B. analyzed the data and drafted the manuscript, which was reviewed and edited by all other authors. C. B. and M. V. carried out the meta-analysis. All authors approved the final manuscript prior to submission.

***Acknowledgments.*** The authors acknowledge the Bill & Melinda Gates Foundation, which funds enteric fever research carried out by our group.

***Financial support.*** C. B. is a Rhodes scholar funded by the Rhodes Trust. M. V. is funded by a National Institutes of Health Research Doctoral Research Fellowship (DRF-2015-08-048).

***Potential conflicts of interest.*** A. J. P. has previously conducted studies on behalf of Oxford University funded by vaccine manufacturers, but currently does not undertake industry-funded clinical trials. A. J. P. also chairs the JCVI and is a member of SAGE. All other authors report no potential conflicts. No reported conflicts of interest. All authors have submitted the ICMJE Form for Disclosure of Potential Conflicts of Interest. Conflicts that the editors consider relevant to the content of the manuscript have been disclosed.
