# Supplementary figures and images for: An Appraisal of the Clinical Features of Pediatric Enteric Fever: Systematic Review and Meta-analysis of the Age-Stratified Disease Occurrence
Source: Clin Infect Dis. 2017 Mar 27;64(11):1604–11. doi: 10.1093/cid/cix229 (PMC5434381; doi:10.1093/cid/cix229)

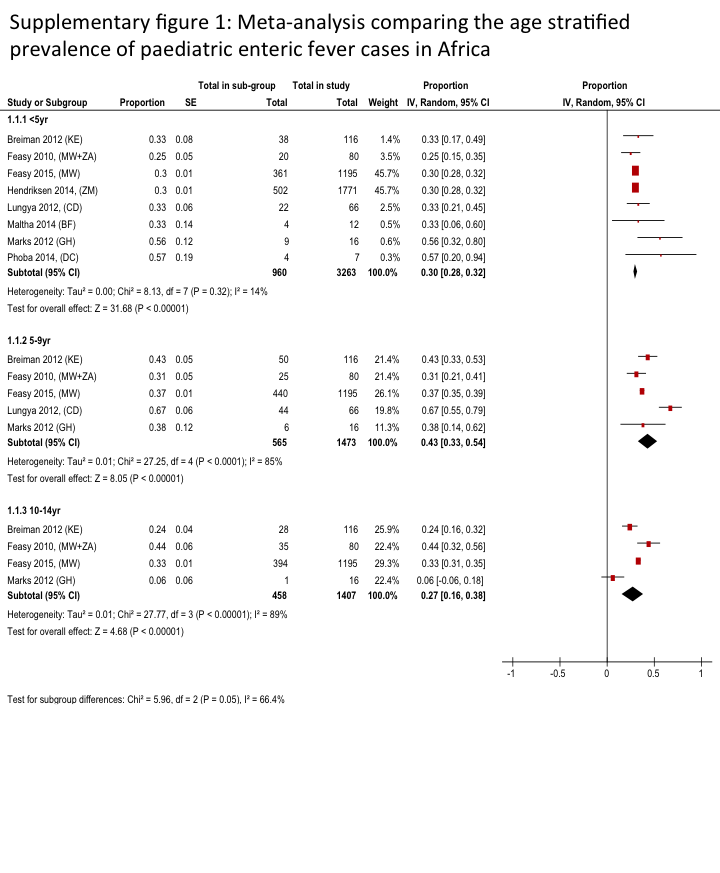

Supplement: Supplememtary_1 [file cix229_suppl_Supplememtary_1.png]

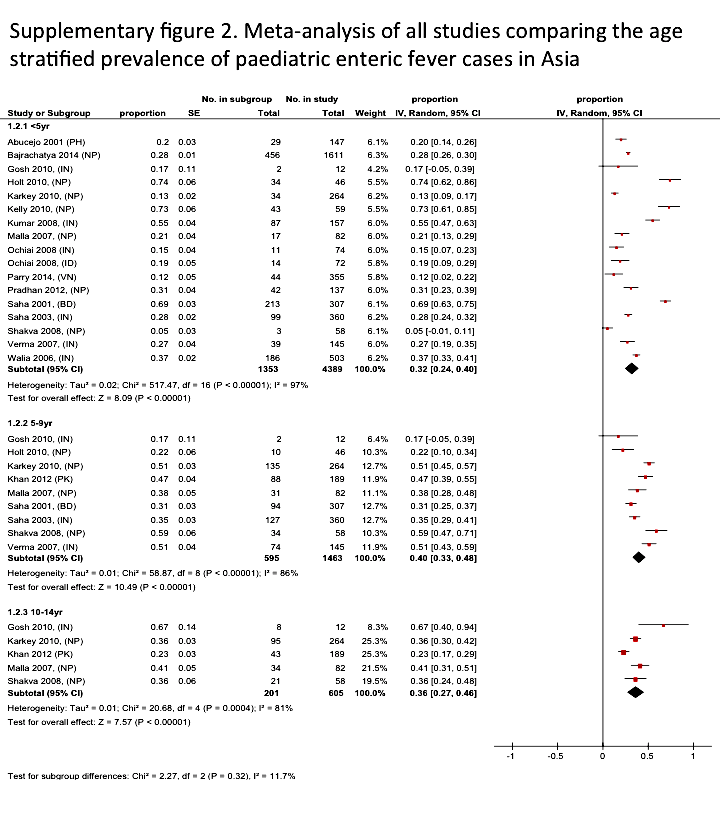

Supplement: Supplementary_2 [file cix229_suppl_Supplementary_2.png]

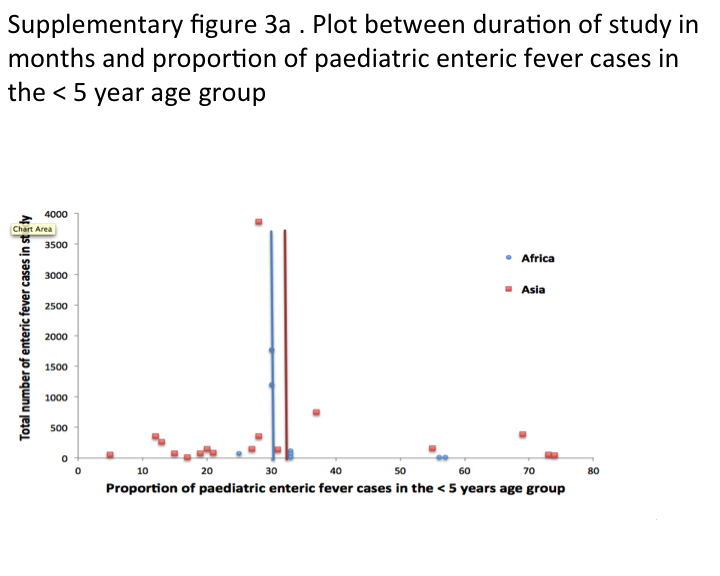

Supplement: Supplementary_3a [file cix229_suppl_Supplementary_3a.png]

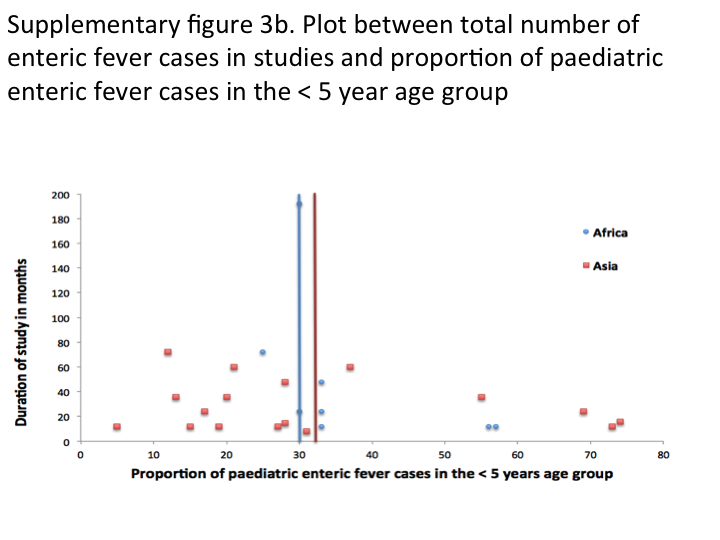

Supplement: Supplementary_3b [file cix229_suppl_Supplementary_3b.png]

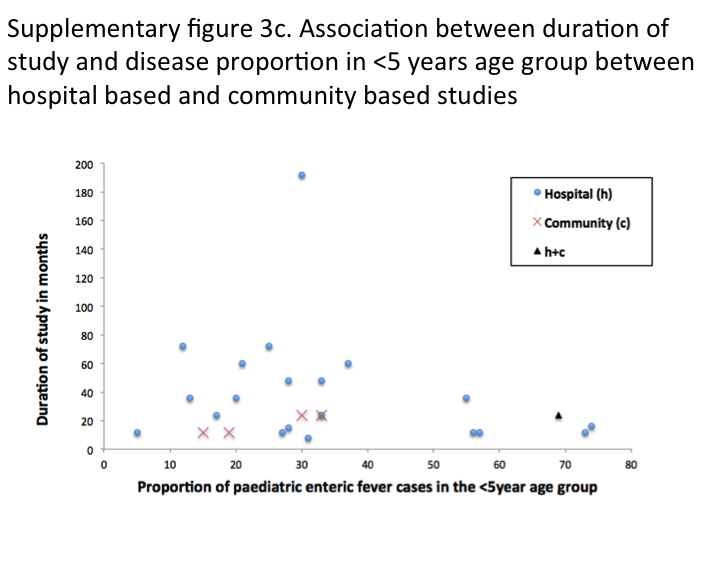

Supplement: Supplementary_3c [file cix229_suppl_Supplementary_3c.png]
