# Supplementary material for: An Appraisal of the Clinical Features of Pediatric Enteric Fever: Systematic Review and Meta-analysis of the Age-Stratified Disease Occurrence
Source: Clin Infect Dis. 2017 Mar 27;64(11):1604–11. doi: 10.1093/cid/cix229 (PMC5434381; doi:10.1093/cid/cix229)
Supplement: Supplementary-material_2 [file cix229_suppl_Supplementary-material_2.docx]

**Supplementary Material:**

**Data input and analysis (continued)**

We conducted this systematic review and meta-analysis and reported our findings in accordance with the GATHER statement^101^ as this statement is designed to report health estimates. The proportion of paediatric enteric fever cases was determined for each age-category (<5 years, 5-9 years and 10-14 years), with the denominator being the number of cases aged 0 to 14 years. To remove the possibility of bias due to different age-distributions in the population for different countries, the proportion of paediatric enteric fever cases in each age category were standardized based on age-specific population proportions for the relevant country^89^. African and Asian studies were meta-analysed separately (Figures 1 & 2, Supplementary Figures 1&2). Sensitivity analyses were conducted which excluded small studies of less than 50 participants. All meta-analyses were conducted using RevMan version 5.3^90^ with random effects models^91^. There were 16 studies from Asia^15–20,29,36,38,92–98^ and 8 from Africa^8–12,35,99,100^ included in the initial meta-analyses of which 5 were excluded from the sensitivity analysis^10,11,35,38,94^

| **Supplementary table 1. Summary of included studies in the meta-analysis** | | | | | | | |
| --- | --- | --- | --- | --- | --- | --- | --- |
|  | **Author (year)** | **Country** | **Study design** | | **Study Setting** | **Period of Data Collection** | **Sample size** |
| **African Studies** | | | | | | | |
| 1. | Breiman (2012)^99^ | Kenya | Prospective | Population based surveillance | Community based | 2007-2009 | 116 |
| 2. | Feasy (2015)^9^ | Malawi | Prospective | Passive surveillance | Hospital based | 1998-2014 | 1195 |
| 3. | Hendriksen (2014)^8^ | Zambia | Retrospective | Cross-sectional study during an outbreak | Community based | 2010-2012 | 1771 |
| 4. | Lungya (2012)^100^ | Democratic republic of Congo | Prospective | Laboratory surveillance study | Hospital based  (7 centers) | 2007-2011 | 66 |
| 5. | Feasy (2010)^12^ | Malawi and South Africa | Retrospective (Malawi)  Laboratory Surveillance (South Africa) | Hospital based | Hospital based | 1998-2004 (Malawi)  2003-2004 (South Africa) | 80 |
| 6. | Maltha (2014)^35^ | Burkina Faso | Prospective | Invasive bacterial surveillance | Hospital based | 2008-2009 | 12 |
| 7. | Marks (2010)^10^ | Ghana | Prospective |  | Hospital based | 2007-2008 | 16 |
| 8. | Phoba (2014)^11^ | Democratic republic of Congo | Prospective | Active surveillance | Hospital based | 2011-2012 | 11 |
| **Asian Studies** | | | | | | | |
| 1 | Abucejo (2001)^92^ | Philippines | Prospective | Laboratory surveillance | Hospital based | 1994-1997 | 147 |
| 2 | Bajrachatya (2014)^93^ | Nepal | Retrospective | Retrospective chart review | Hospital based (11 centers) | 2008-2012 | 3857 |
| 3 | Gosh (2010)^38^ | India | Prospective | Passive surveillance | Hospital based | 2003-2005 | 12 |
| 4 | Holt (2010)^94^ | Nepal | Prospective | Invasive bacterial (passive) surveillance | Hospital based | 2005-2006 | 46 |
| 5 | Karkey (2010)^15^ | Nepal | Retrospective | Invasive bacterial (passive) surveillance | Hospital based | 2005-2008 | 264 |
| 6 | Kelly (2010)^19^ | Nepal | Retrospective | Invasive bacterial (passive) surveillance | Hospital based | 2005-2006 | 59 |
| 7 | Kumar (2008)^18^ | India | Prospective | Passive surveillance | Hospital based | 1999, 2002, 2005 | 157 |
| 8 | Malla (2007)^95^ | Nepal | Retrospective | Chart review | Hospital based | 2000-2005 | 82 |
| 9 | Ochiai (2008)^29^ | India | Prospective | Active Surveillance? | Community baseAfri | 2003-2004 | 74 |
|  | Ochiai (2008)^29^ | Indonesia | Prospective | Active Surveillance? | Community based | 2003-2004 | 72 |
| 10 | Parry (2014)^16^ | Vietnam | Retrospective | Chart review | Hospital based (2 centers) | 1993-1995, 1997-1999 | 355 |
| 11 | Pradhan (2012)^36^ | Nepal | Prospective | Invasive bacterial surveillance | Hospital based | April-August 2006,  December-February 2007 | 137 |
| 12 | Saha (2001)^20^ | Bangladesh | Prospective | Passive surveillance | Community + hospital based | 1998-1999 | 391 |
| 13 | Saha (2003)^96^ | India | Prospective | Passive Surveillance | Hospital based | 1990-2002 | 360 |
| 14 | Shakva (2008)^17^ | Nepal | Prospective | Passive surveillance | Hospital based | 2006-2007 | 58 |
| 15 | Verma (2007)^97^ | India | Prospective |  | Hospital based | 2004-2005 | 145 |
| 16 | Walia (2006)^98^ | India | Retrospective | Chart review | Hospital based | 1999-2003 | 750 |

| **Supplementary table 2. GATHER checklist** | | |
| --- | --- | --- |
| **Context and Design** | | |
| 1. | Definition of the indicator(s) estimated, populations (including age, sex, geographic region) for which estimates are made, and time period(s) for which estimates were made. | ✓ |
| **Data Inputs** | | |
| 2 | Describe the data identification and access strategy | ✓ |
| 3 | Define the inclusion/exclusion criteria, including ad‐hoc exclusions (e.g., outliers) | ✓ |
| 4 | Identify and describe types of input data that have potentially important biases | ✓ |
| 5 | Provide key characteristics of input data sources | ✓ |
| 6. | Describe and give sources for other inputs (for data not synthesized for study) | ✓ |
| 7 | Make all data inputs available, with the exception for confidential data, for which contact names/institutions are made available | ✓ |
| **Analytic Methods** | | |
| 8 | Provide a [conceptual] overview of analytic steps | ✓ |
| 9 | Detailed description of analytic steps. | ✓ |
| 10 | Model building (model development process and model selection) | NA – Random effects meta-analysis |
| 11 | Model performance (including sensitivity analysis, if relevant) | ✓ |
| 12 | Uncertainty | Confidence Intervals estimated from Random effects meta-analysis |
| 13 | State where computer/statistical code can be accessed | NA |
| **Results** | | |
| 14 | Clearly describe results.  This item includes a series of suggested ‘best practices’ for describing results in a way that is accessible to all users. | ✓ |
| 15 | Provide published estimates in electronic data format | ✓ |
| 16 | Address uncertainty, with recommendation confidence intervals be given. Discuss why uncertainty was not calculated, if not calculated. |  |
| **Discussion** | | |
| 17 | Discuss estimates in view of existing evidence (e.g., other estimates), and if updating previous set of estimates, discuss reasons for changes in estimates | NA |
| 18 | Discuss limitations of the estimates | ✓ |
| 19 | List the funding sources for the work | ✓ |

**Supplementary material legends.**

**1. Supplementary Table 1. Summary of studies included in the systematic review and meta-analysis**

**2. Supplementary table 2. GATHER checklist**

**3. Supplementary Figure 1. Meta-analysis of all studies comparing the age stratified burden of paediatric enteric fever cases from Africa**

Studies in the meta-analysis are identified based on author name, year of publication with country codes in parenthesis (country codes used are those supplied by the International Standardization Organisation).

SE: standard error; IV: inverse variance; Proportion: (Number of children in age group / number of children in the study aged 0–14 years). Proportions are standardized according to population age-distributions for the three age categories. The analysis was done using random effects model.

**4. Supplementary Figure 2. Meta-analysis of all studies comparing the age stratified burden of paediatric enteric fever cases from Asia**

Studies in the meta-analysis are identified based on author name, year of publication with country codes in parenthesis (country codes used are those supplied by the International Standardization Organisation).

SE: standard error; IV: inverse variance; Proportion: (Number of children in age group / number of children in the study aged 0–14 years). Proportions are standardized according to population age-distributions for the three age categories. The analysis was done using random effects model.

**5. Supplementary Figure 3. To determine possible sources of heterogeneity in the < 5 years age group.**

**a. Plot between duration of study in months and proportion of paediatric enteric fever cases in the < 5 years age group**

Vertical lines indicate the mean proportion of paediatric enteric fever in the <5 year age group in Africa (30) and Asia (32) respectively

**b. Plot between total number of enteric fever cases in studies and proportion of paediatric enteric fever cases in the < 5 years age group**

Vertical lines indicate the mean proportion of paediatric enteric fever in the <5 year age group in Africa (30) and Asia (32) respectively

**c. Association between duration of study and disease proportion in <5 years age group between hospital based and community-based studies**

**References: The references below are in a continuation of those cited in the main manuscript**

41 Bhutta ZA. Impact of age and drug resistance on mortality in typhoid fever. *Arch Dis Child* 1996; **75**: 214–7.

42 Who. Background document : The diagnosis, treatment and prevention of typhoid fever. *Commun Dis Surveill Response Vaccines Biol* 2003. www.who.int/vaccines-documents/.

43 Topley JM. Mild typhoid fever. *Arch Dis Child* 1986; **61**: 164–7.

44 Arora_1991_Plant-Science. .

45 Rasaily R, Dutta P, Saha MR, Mitra U, Lahiri M, Pal SC. Multi-drug resistant typhoid fever in hospitalised children. Clinical, bacteriological and epidemiological profiles. *Eur J Epidemiol* 1994; **10**: 41–6.

46 Shwe TN, Nyein MM, Yi W, Mon A. Blood culture isolates from children admitted to Medical Unit III, Yangon Children’s Hospital, 1998. *Southeast Asian J Trop Med Public Heal* 2002; **33**: 764–71.

47 Bhan MK, Bahl R, Bhatnagar S. Typhoid and paratyphoid fever. *Lancet* 2015; **366**: 749–62.

48 Butler T, Islam A, Kabir I, Jones PK. Patterns of morbidity and mortality in typhoid fever dependent on age and gender: review of 552 hospitalized patients with diarrhea. *Rev Infect Dis* 1991; **13**: 85–90.

49 Ahmad KA, Khan LH, Roshan B, Bhutta Z a. Factors associated with typhoid relapse in the era of multiple drug resistant strains. *J Infect Dev Ctries* 2011; **5**: 727–31.

50 Nasir AA, Abdur-Rahman LO, Adeniran JO. Predictor of mortality in children with typhoid intestinal perforation in a Tertiary Hospital in Nigeria. *Pediatr Surg Int* 2011; **27**: 1317–21.

51 Onen A, Dokucu AI, Cigdem MK, Ozturk H, Otcu S, Yucesan S. Factors effecting morbidity in typhoid intestinal perforation in children. *Pediatr Surg Int* 2002; **18**: 696–700.

52 Alexander L, Allen S, Bindoff NL. Summary for Policymakers. *Eff Br mindfulness Interv acute pain Exp An Exam Individ Differ* 2013; **1**: 1–36.

53 Malik AS. Complications of bacteriologically confirmed typhoid fever in children. *J Trop Pediatr* 2002; **48**: 102–8.

54 Verma M. Brief Reports Enteric Fever Below 2 Years of Age. 1996; **33**: 229–30.

55 Huang DB, DuPont HL. Problem pathogens: extra-intestinal complications of Salmonella enterica serotype Typhi infection. *Lancet Infect Dis* 2005; **5**: 341–8.

56 Khair AM, Elmagrabi D. Febrile Seizures and Febrile Seizure Syndromes: An Updated Overview of Old and Current Knowledge. *Neurol Res Int* 2015; **2015**: 1–7.

57 Hoffman SL, Punjabi NH, Kumala S, *et al.* Reduction of mortality in chloramphenicol-treated severe typhoid fever by high-dose dexamethasone. *N Engl J Med* 1984; **310**: 82–8.

58 Song J, Gao X, Galán JE. Structure and function of the Salmonella Typhi chimaeric A(2)B(5) typhoid toxin. *Nature* 2013; **499**: 350–4.

59 Heitmann D, Ziehr H, Müthing J. Large scale purification of gangliosides G(M3)(Neu5Ac) and G(M3)(Neu5Gc) by trimethylaminoethyl-fractogel high-performance liquid chromatography. *J Chromatogr B Biomed Appl* 1998; **710**: 1–8.

60 Uysal H, Karademir A, Kilinc M, Erturk O. Salmonella encephalopathy with seizure and frontal intermittent rhythmic delta activity. *Infection* 2001; **29**: 103–6.

61 Desai JD, Toczek MT, Mitchell WG. Frontal intermittent rhythmic delta activity (FIRDA): is there a clinical significance in children and adolescents? *Eur J Paediatr Neurol* 2012; **16**: 138–41.

62 Balasubramanian S, Shivbalan S, Miranda PKL. Pseudotumour cerebri as an unusual manifestation of typhoid. Ann. Trop. Paediatr. 2003; **23**: 223–4.

63 Bhatt GC, Dewan V, Dewan T, Yadav TP. Pseudotumour cerebri with multiple cranial nerve palsies in enteric fever. Indian J. Pediatr. 2014; **81**: 196–7.

64 Moodley M, Coovadia HM. Benign intracranial hypertension in typhoid fever. A case report. *S Afr Med J* 1990; **78**: 608–9.

65 Chow CB, Wang PS, Leung NK. Typhoid fever in Hong Kong children. *Aust Paediatr J* 1989; **25**: 147–50.

66 Hanel RA, Araujo JC, Antoniuk A, da Silva Ditzel LF, Flenik Martins LT, Linhares MN. Multiple brain abscesses caused by Salmonella typhi: case report. *Surg Neurol* 2000; **53**: 86–90.

67 Incecik F, Herguner MO, Mert G, Alabaz D, Altunbasak S. Acute cerebellar ataxia associated with enteric fever in a child: a case report. *Turk J Pediatr* 2013; **55**: 441–2.

68 Okunola PO, Ofovwe GE, Abiodun MT, Azunna CP. Superior sagittal sinus thrombosis complicating typhoid Fever in a teenager. *Case Rep Pediatr* 2012; **2012**: 201203.

69 Rajeshwari K, Yadav S, Puri RK, Khanijo CM, Sethi Y. Cerebritis in typhoid fever. *Indian Pediatr* 1995; **32**: 1305–7.

70 Singh RR, Chaudhary SK, Bhatta NK, Khanal B, Shah D. Clinical and etiological profile of acute febrile encephalopathy in eastern Nepal. *Indian J Pediatr* 2009; **76**: 1109–11.

71 Ozen H, Cemeroglu P, Ecevit Z, Secmeer G, Kanra G. Unusual neurologic complications of typhoid fever (aphasia, mononeuritis multiplex, and Guillain-Barre syndrome): a report of two cases. *Turk J Pediatr* 1993; **35**: 141–4.

72 Mehndiratta S, Rajeshwari K, Dubey AP. Guillain-Barre syndrome as a complication of typhoid fever in a child. Neurol. India. 2012; **60**: 433–5.

73 May W, Senitiri I. Guillain-Barre syndrome associated with typhoid fever. A case study in the Fiji Islands. *Pac Health Dialog* 2010; **16**: 85–8.

74 Kapoor K, Jain S, Jajoo M, Talukdar B. A rare neurological complication of typhoid fever: Guillain-Barre’ syndrome. *J Pediatr Neurosci* 2014; **9**: 148–9.

75 Donoso R. Guillain-Barre syndrome following typhoid fever/letter. *Ann Neurol* 1988; **23**: 627.

76 Datta V, Sahare P, Chaturved P. Guillain-Barre syndrome as a complication of enteric fever. *J Indian Med Assoc* 2004; **102**: 172–3.

77 Chanmugam D, Waniganetti A. Guillain-Barre syndrome associated with typhoid fever. *Br Med J* 1969; **1**: 95–6.

78 Berger JR, Ayyar DR, Kaszovitz B. Guillain-Barre syndrome complicating typhoid fever. Ann. Neurol. 1986; **20**: 649–50.

79 Aldrey JM, Fernandez-Rial A, Lopez-Gonzalez FJ, Doval JC, de la Fuente-Fernandez R. Guillain-Barre syndrome as first manifestation of typhoid fever. *Clin Infect Dis* 1999; **28**: 1171–2.

80 Malik a S, Malik RH. Typhoid fever in Malaysian children. *Med J Malaysia* 2001; **56**: 478–90.

81 Shetty AK, Mital SR, Bahrainwala AH, Khubchandani RP, Kumta NB. Typhoid hepatitis in children. *J Trop Pediatr* 1999; **45**: 287–90.

82 Ali G, Kamili MA, Rashid S, Mansoor A, Lone BA, Allaqaband GQ. Spontaneous splenic rupture in typhoid fever. *Postgrad Med J* 1994; **70**: 513–4.

83 Julia J, Canet JJ, Lacasa XM, Gonzalez G, Garau J. Spontaneous spleen rupture during typhoid fever. *Int J Infect Dis* 2000; **4**: 108–9.

84 Nesbitt A, Mirza NB. Salmonella septicaemias in Kenyan children. *J Trop Pediatr* 1989; **35**: 35–9.

85 Lai X-H, Xu Y, Chen X-M, Ren Y. Macrophage cell death upon intracellular bacterial infection. *Macrophage* 2015; : 1–11.

86 Ganesh R, Janakiraman L, Vasanthi T, Sathiyasekeran M. Profile of typhoid fever in children from a tertiary care hospital in Chennai-South India. *Indian J Pediatr* 2010; **77**: 1089–92.

87 Butler T, Islam MR, Bardhan PK. The leukemoid reaction in shigellosis. *Am J Dis Child* 1984; **138**: 162–5.

88 Azim T, Qadri F, Ahmed S, *et al.* Lipopolysaccharide-specific antibodies in plasma and stools of children with Shigella-associated leukemoid reaction and hemolytic-uremic syndrome. *Clin Diagn Lab Immunol* 1996; **3**: 701–5.

89 United N. World Population Prospects The 2015 Revison. *Dep Econ Soc Aff* 2015; **53**: 1689–99.

90 The Nordic Cochrane Centre, The Cochrane Collaboration. Review Manager (RevMan) [Computer program]. Version 5.3. Copenhagen: 2014.

91 DerSimonian R, Laird N. Meta-analysis in clinical trials. *Control Clin Trials* 1986; **7**: 177–88.

92 Abucejo PE, Capeding MR, Lupisan SP, Arcay J. BLOOD CULTURE CONFIRMED TYPHOID FEVER IN A PROVINCIAL HOSPITAL IN THE PHILIPPINES. 2001; **3**: 3–8.

93 Bajracharya D, Khan MI, Pach A, *et al.* 25 Years after Vi typhoid vaccine efficacy study, typhoid affects significant number of population in Nepal. *PLoS One* 2014; **9**: 1–4.

94 Holt KE, Baker S, Dongol S, *et al.* High-throughput bacterial SNP typing identifies distinct clusters of Salmonella Typhi causing typhoid in Nepalese children. *BMC Infect Dis* 2010; **10**: 144.

95 Malla, T. Malla, KK. Thapalial, A. Shaw C. Enteric Fever: A retrospective 6-year analysis of 82 paediatric cases in a teaching hospital. *Kathmandu Univ Med J* 2007; **5**: 181–7.

96 Saha MR, Palit A, Chatterjee NS, Dutta P, Mitra U, Bhattacharya SK. A prospective study of phage types & biotypes of Salmonella enterica serotype Typhi isolated from hospitalized children in Kolkata, India. *Indian J Med Res* 2003; **117**: 201–4.

97 Verma M, Parashar Y, Singh A, Kamoji R. Current pattern of enteric fever: A prospective clinical and microbiological study. *J Indian Med Assoc* 2007; **105**: 582–91.

98 Walia M, Gaind R, Paul P, Mehta R, Aggarwal P, Kalaivani M. Age-related clinical and microbiological characteristics of enteric fever in India. *Trans R Soc Trop Med Hyg* 2006; **100**: 942–8.

99 Breiman RF, Cosmas L, Njuguna H, *et al.* Population-based incidence of typhoid fever in an urban informal settlement and a rural area in Kenya: implications for typhoid vaccine use in Africa. *PLoS One* 2012; **7**: e29119.

100 Lunguya O, Lejon V, Phoba M-F, *et al.* Salmonella typhi in the democratic republic of the congo: fluoroquinolone decreased susceptibility on the rise. *PLoS Negl Trop Dis* 2012; **6**: e1921.
